# Nomogram for predicting the survival of gastric adenocarcinoma patients who receive surgery and chemotherapy

**DOI:** 10.1186/s12885-019-6495-2

**Published:** 2020-01-06

**Authors:** Chao-Yang Wang, Jin Yang, Hao Zi, Zhong-Li Zheng, Bing-Hui Li, Yang Wang, Zheng Ge, Guang-Xu Jian, Jun Lyu, Xiao-Dong Li, Xue-Qun Ren

**Affiliations:** 1 Department of General Surgery, Huaihe Hospital of Henan University, Kaifeng, Henan China; 2 Institute of Evidence-Based Medicine and knowledge translation, Henan University, Kaifeng, Henan China; 3 Clinical Research Center, The First Affiliated Hospital of Xi’an Jiaotong University, Xi’an, Shaanxi China; 4 School of Public Health, Xi’an Jiaotong University Health Science Center, Xi’an, Shaanxi China; 5 Department of ICU, Huaihe Hospital of Henan University, Kaifeng, Henan China; 6 Department of Urology, Huaihe Hospital of Henan University, Kaifeng, Henan China

**Keywords:** Gastric adenocarcinoma, Nomogram, Surgery, Chemotherapy, SEER, Disease-specific survival

## Abstract

**Background:**

Surgery is the only way to cure gastric adenocarcinoma (GAC), and chemotherapy is the basic adjuvant management for GAC. A significant prognostic nomogram for predicting the respective disease-specific survival (DSS) rates of GAC patients who receive surgery and chemotherapy has not been established.

**Objective:**

We were planning to establish a survival nomogram model for GAC patients who receive surgery and chemotherapy.

**Methods:**

We identified 5764 GAC patients who had received surgery and chemotherapy from the record of Surveillance, Epidemiology, and End Results (SEER) database. About 70% (*n* = 4034) of the chosen GAC patients were randomly assigned to the training set, and the rest of the included ones (*n* = 1729) were assigned to the external validation set. A prognostic nomogram was constructed by the training set and the predictive accuracy of it was validated by the validation set.

**Results:**

Based on the outcome of a multivariate analysis of candidate factors, a nomogram was developed that encompassed age at diagnosis, number of regional lymph nodes examined after surgery, number of positive regional lymph nodes**,** sex**,** race, grade, derived AJCC stage, summary stage, and radiotherapy status. The C-index (Harrell’s concordance index) of the nomogram model was some larger than that of the traditional seventh AJCC staging system (0.707 vs 0.661). Calibration plots of the constructed nomogram displayed that the probability of DSS commendably accord with the survival rate. Integrated discrimination improvement (IDI) revealed obvious increase and categorical net reclassification improvement (NRI) showed visible enhancement. IDI for 3-, 5- and 10- year DSS were 0.058, 0.059 and 0.058, respectively (*P* > 0.05), and NRI for 3-, 5- and 10- year DSS were 0.380 (95% CI = 0.316–0.470), 0.407 (95% CI = 0.350–0.505), and 0.413 (95% CI = 0.336–0.519), respectively. Decision curve analysis (DCA) proved that the constructed nomogram was preferable to the AJCC staging system.

**Conclusion:**

The constructed nomogram supplies more credible DSS predictions for GAC patients who receive surgery and chemotherapy in the general population. According to validation, the new nomogram will be beneficial in facilitating individualized survival predictions and useful when performing clinical decision-making for GAC patients who receive surgery and chemotherapy.

## Background

Gastric cancer (GC) is a common type of cancer worldwide, with more than 1 million new cases in 2018, and it causes nearly 800,000 deaths. The GC causing deaths take part in one twelfth global deaths. Thus, GC is the fifth most usually diagnosed cancer and makes the third predominant cause of cancer-related deaths [1]. GC has a routine appearance of adenocarcinoma in 90% of cases, with gastric adenocarcinoma (GAC) being the most-common subtype of GC [2]. The incidence of GC varies between regions, with approximate 70% of cases taking place in developing countries [3]. The incidence rate in men is two fold higher than that in women. Among men, GC is the most important diagnosed type of cancer and the predominant cause of cancer-related deaths in some countries in western Asian, including Iran, Turkmenistan, and Kyrgyzstan. The incidence rates of GC are also obviously elevated in countries in Eastern Asia, such as Mongolia, Japan, and the Republic of Korea [1].

The main treatment modality for locally advanced GC is stomach resection by surgery, and complete resection is the essential treatment for curing locally advanced GC. However, while complete resection by surgery can eliminate the cancer that is visible in the surgical field [4], cancer recurrence remains possible since complete resection cannot extinguish any micrometastatic cancer cells that exist outside of the surgical field. Such unseen cancer cells inevitably reproduce to become a lump that can be diagnosed on imageological examinations or physical examinations, corresponding to recurrence [5]. The objective of adjuvant therapy is to extinguish micrometastatic tumor cells before and/or after surgery in order to increase the probability of a good survival outcome for the cancer patient. The timing to perform chemotherapy is different in the world. In the European Union and the USA, preoperative chemotherapy is advocated while postoperative adjuvant chemotherapy is encouraged in Asia [6].

It is popular to use nomograms for cancer prognoses because nomograms simplify complex statistical predictive models containing large quantity of factors to a single brief numerical estimate model to predict the probability of an event. Such as death or recurrence of cancer. A nomogram is specific to an individual patient [7, 8]. Nomogram is easily mastered graphical interfaces to inform clinical decision-making. Nomogram have in general been generally used as graphical representations of complex mathematical formulas. Nomograms combine some independent factors to get a statistical prognostic model. And the model was estimated the prognosis in multiple malignancies [9].

Some nomograms for the survival prognosis of GC or GAC have been reported [10–15]. However, no nomograms are available for the 10-year survival prognosis of GAC patients who have received surgery and chemotherapy. In the current study we planed to construct a survival nomogram to predicting the survival of GAC patients who receive surgery and chemotherapy.

## Methods

### Patients

The Surveillance, Epidemiology, and End Results (SEER) database contains nearly 30% of the total US population. It is composed of 18 registries form different cities containing important clinical information on patients in the US who suffered from tumors. We obtained clinical information on GAC patients from the SEER database that could be detailed analyses of survival in GAC. This study performed a retrospective review of all GAC patients in the SEER database who had received surgery and chemotherapy between 2004 and 2015. In order to assess the effect of lymph node status, patients with enough information about the number of regional lymph nodes examined (RNE) were selected in the current study. Finally, accord to the inclusion criteria, a total of 5764 GAC patients were selected as the primary cohort. Approximately 70% (*n* = 4034) of these patients were randomly assigned to the training set, and the rest 1729 patients were defined as the external validation set.

The inclusion criteria for GAC patients in the current study were described as follows:

1. Site and morphology according to International Classification of Diseases for Oncology (ICD-O-3) histology/behavior code 8140/3, 8141/3, 8142/3, 8143/3, 8144/3, 8146/3, 8147/3, 8149/3, 8213/3, 8262/3, 8263/3, 8290/3, 8310/3, 8322/3, 8323/3, 8325/3, 8330/3, 8331/3, 8332/3, or 8333/3.
2. Site and morphology, according to ICD-O-3 primary site code C16.0, C16.1, C16.2, C16.3, C16.4, C16.5, C16.6, C16.7, C16.8, or C16.9.
3. Known cause of death and known survival period after the diagnosis.
4. Received either local or major primary tumor resection.
5. Received chemotherapy.

The exclusion criteria for GAC patients in the current study were as follows:

1. GAC was not the only primary cancer diagnosed.
2. Unknown AJCC stage.
3. Unknown TNM stage.
4. Unknown lymph node status.

### Ethical approval

All the date from SEER database are de-identified before being released to the public and so cases extracted do from the SEER database do not contain any personally identifying information. Since the data are available to all the researchers when they signed to obey the data use agreement, no ethical approval was required for this study.

### Data collection

The potential factors associated with the survival of GAC patients who receive surgery and chemotherapy were identified by obtaining information on the clinicopathological characteristics of these patients such as the age at diagnosis, RNE, number of positive regional lymph nodes (RNP), sex, race, grade, derived AJCC stage, summary stage, and radiotherapy status. The end point of this study was the disease-specific survival (DSS) rate. DSS was defined as the time period from surgery to cancer-caused death or the last follow-up. DSS was calculated and survival curves were produced using the Kaplan-Meier method and the outcome of the curves were validated by the log-rank test.

### Statistical analysis

Age, RNE, and RNP were continuous variables in this study. Age and RNE are in accordance with normal distribution and they are expressed as the form of mean ± SD values, while RNP is expressed as median and interquartile-range values since it dose not follow the law of the normal distribution. The rest variables are categorical and are presented as percentages. Independent factors predicting the survival time were determined using the Kaplan-Meier and Cox proportional-hazards models [16]. Variables that were significant were further identified by a multivariate Cox proportional-hazards model via backward stepwise analysis.

A nomogram to predict the 3-, 5-, and 10-year DSS rates was constructed using the results of the multivariate analyses. The predictive accuracy of the constructed nomogram was estimated using Harrell’s concordance index (C-index) and the area below the time-dependent receiver operating characteristic curve (AUC). Calibration was assessed graphically by plotting the relationship between the predicted probability and the actual outcome using the Hosmer goodness-of-fit test [17]. The integrated discrimination improvement (IDI) and the net reclassification improvement (NRI) were calculated to evaluate the improved advantage in the predictive accuracy of the new prediction model [18].. Finally, decision-curve analysis (DCA) was employed to evaluate the clinical applicability of the constructed nomogram by quantifying the net improved benefits at various threshold probabilities [19].

All statistical analyses were carried out using SPSS software (version 24.0, SPSS, Chicago, IL, USA) and R software. A two-sided *P* value of ≤0.05 was regarded as existing statistical significance.

## Results

### Patient baseline characteristics

After selection in SEER database according to the inclusion and exclusion criteria, 5764 patients who received surgery and chemotherapy were identified. Approximate 70% of them were grouped into Training set randomly, while the rest were randomly selected into the validation set. In the training set, the age at diagnosis was 62.8 ± 11.6 years (range 22–92 years). There were 2905(72%) male patients and 1129 (28%) female ones. These patients were predominantly white (*n* = 2805, 69.5%), while 516 of them were black (12.8%) and 713 were of other races (17.7%). Their marital status comprised 2811 married patients (69.5%), 475 patients (11.8%) who were single or living with a domestic partner, and 748 patients (18.5%) were divorced or separated or widowed (DSW). The primary sites of GAC in 1698 patients (42.1%) were located in cardia, 1392 patients (34.5%)were located in pylorus, and 944 patients (23.4%) were located in other part of the stomach or the location were unknown. Poor differentiation (61.3%) was the most-common tumor grade, followed by moderate differentiation (33.5%), well-differentiated (3.2%), and undifferentiated (2.0%). Most patients (56.2%) were categorized as primary T category T2, 27.1% were T3, 8.8% were T1, and 8.0% were T4. About half of the patients (53.2%) were categorized as primary N category N1, 22.9% were N0, 18.0% were N2, and 5.9% were N3, while 89.6% were categorized as primary M category M0 and 11.4% were M1. Regional cancer (72.3%) was the most-common tumor summary stage, followed by localized cancer (14.0%) and distant cancer (13.7%). Almost half of the patients (44.6%) had the radiation record**.** The RNE was 18.2 ± 11.9 (range 1–87), and the median RNP was 2 (range 0–79). There were 1333 (33.0%), 1078 (26.7%), 846 (21%), and 777 (19.3%) patients categorized as AJCC stages II, III, I, and IV, respectively.

Patients in the validation set showed similar characteristics to those in the training set. The comprehensive clinicopathological characteristics of the GAC patients involved in the training and validation sets are displayed in Table 1.

**Table 1:** Patient characteristics in the study

Characteristics	Training set (*n* = 4034)		Validation set (*n* = 1729)	
	n	%	n	%
age (years)				
Mean	62.8. ± 11.6		62.5 ± 11.4	
Range	22–92		17–94	
Sex				
Male	2905.0	72.0	1285.0	74.3
Female	1129.0	28.0	444.0	25.7
Race				
White	2805.0	69.5	1215.0	70.3
Black	516.0	12.8	207.0	12.0
Others	713.0	17.7	307.0	17.7
Marital				
Married	2811.0	69.7	1191.0	68.9
Single/Domestic Partner	475.0	11.8	239.0	13.8
DWS	748.0	18.5	299.0	17.3
Prime Site				0.0
cardia	1698.0	42.1	756.0	43.7
pylorus	1392.0	34.5	571.0	33.0
others or primary site unknown	944.0	23.4	402.0	23.3
Grade				
Well	131.0	3.2	55.0	3.2
Moderately	1353.0	33.5	583.0	33.7
Poorly	2471.0	61.3	1053.0	60.9
Undifferentiated	79.0	2.0	38.0	2.2
Primary T category				
T1	353.0	8.8	141.0	8.2
T2	2267.0	56.2	984.0	56.9
T3	1093.0	27.1	477.0	27.6
T4	321.0	8.0	127.0	7.3
Primary N category				
N0	925.0	22.9	373.0	21.6
N1	2145.0	53.2	939.0	54.3
N2	728.0	18.0	317.0	18.3
N3	236.0	5.9	100.0	5.8
Primary M category				
M0	3614.0	89.6	1558.0	90.1
M1	420.0	10.4	171.0	9.9
Summary stage				
Localized	565.0	14.0	225.0	13.0
Regional	2915.0	72.3	1281.0	74.1
Distal	554.0	13.7	223.0	12.9
Radiation recode				0.0
Yes	1800.0	44.6	772.0	44.7
No/Unknown	2234.0	55.4	957.0	55.3
RNE				
mean	18.2 ± 11.9		18.2 ± 11.7	
range	1–87		1–77	
RNP				
median	2		2	
range	0–79		0–51	
AJCC				
I	846.0	21.0	355.0	20.5
II	1333.0	33.0	574.0	33.2
III	1078.0	26.7	477.0	27.6
IV	777.0	19.3	323.0	18.7

*Abbreviations*; *RNE* Number of regional nodes examined, *DSW* divorced & separated &widowed, *RNP* Number of regional nodes positive, *AJCC* American Joint Committee on Cancer

### Nomogram construction

**Fig. 1:**
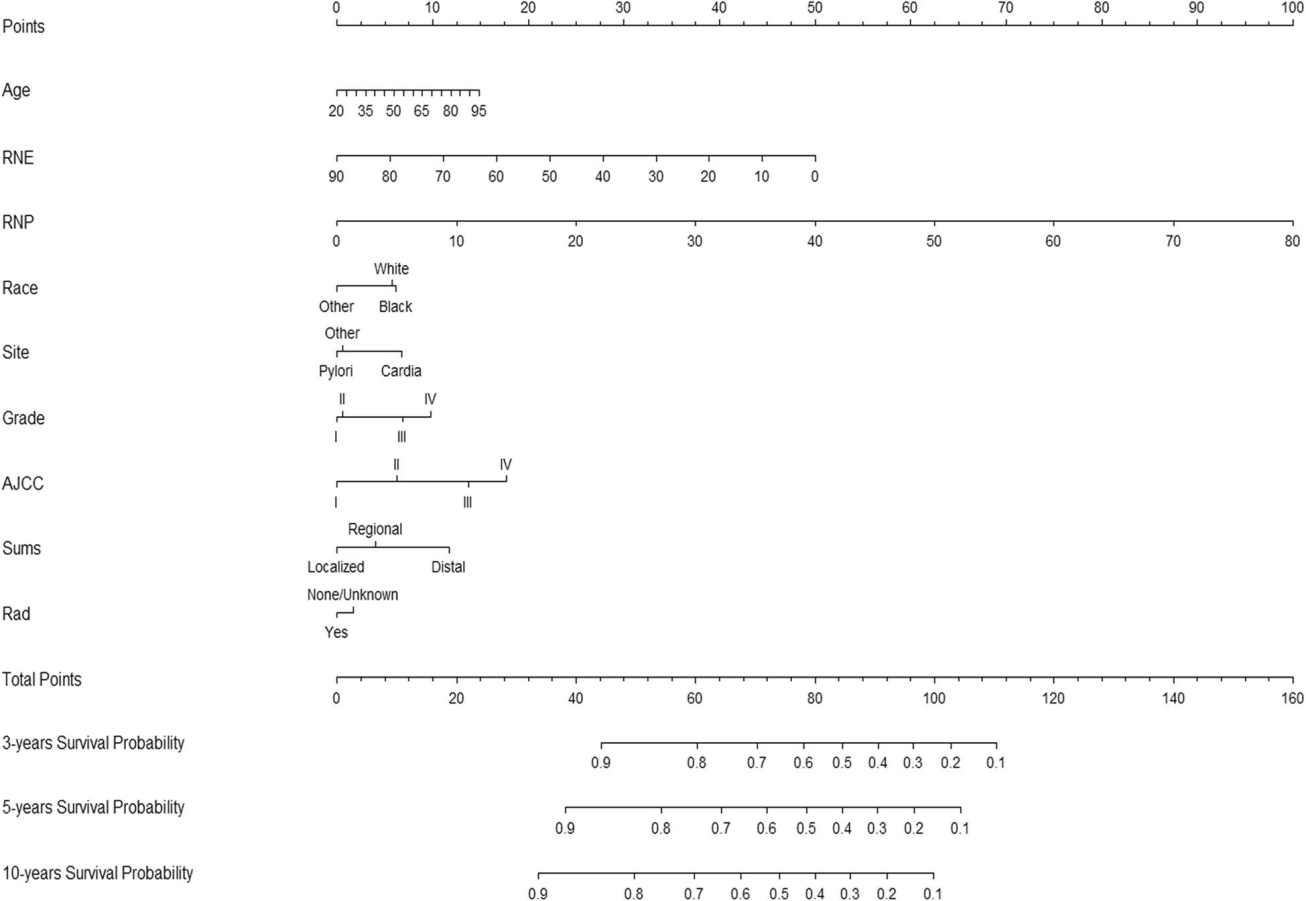
Nomogram predicting 3-,5- and 10-year survival. RNE = Number of regional lymph nodes examined, RNP = number of regional nodes positive, Site = Prime Site, Grade = Differentiation classification, I:Well differentiated, II: Moderately differentiated, III: Poorly differentiated, IV: Undifferentiated, AJCC = Derived AJCC Stage Group, 7thed, Sums = SEER Summary stage 2000, Rad = Radiation recode, Yes: Beam Radiation/ Combination of beam with implants or isotopes/ Other radiation (1973–1987 cases only) /Radiation, NOS method or source not specified/ Radioactive implants/ Radioisotopes, NO:None/Unknown/refused/recommended, unknow if administered

After the multivariable Cox analysis, the outcomes revealed that the age at diagnosis, RNE, RNP, sex, race, grade, summary stage, and radiotherapy status can independently predict the DSS of GAC patients who receive surgery and chemotherapy (Table 2). All of the statistically potential independent risk factors that were related with DSS were incorporated in the prognostic nomogram developed in this study (Fig. 1).

**Table 2:** Selected variables by multivariate Cox regression analysis

Characteristics	HR	95% CI	*p*-value
Age	1.009	1.005–1.013	<0.001
Race			
White	reference		
Black	1.020	0.884–1.175	0.790
Others	0.765	0.668–0.877	<0.001
Prime Site			<0.001
cardia	reference		
pylorus	0.729	0.649–0.818	
others or primary site unknown	0.749	0.660–0.849	
Grade			
Well	reference		
Moderately	1.026	0.743–1.417	0.877
Poorly	1.379	1.004–1.894	0.047
Undifferentiated	1.581	1.026–2.437	0.038
Summary stage			
Localized	reference		
Regional	1.208	0.919–1.587	0.175
Distal	1.726	1.263–2.359	<0.001
Radiation recode			
Yes	reference		
No/Unknown	1.084	0.986–1.192	0.094
RNE	0.974	0.970–0.979	<0.001
RNP	1.06	1.051–1.069	<0.001
AJCC			
I	reference		
II	1.340	1.064–1.687	0.013
III	1.900	1.507–2.397	<0.001
IV	2.285	1.750–2.981	<0.001

*Abbreviations*; *RNE* number of regional nodes examined, *DSW* divorced & separated &widowed, *RNP* number of regional nodes positive, *AJCC* American Joint Committee on Cancer

### Validation of the nomogram for DSS of GAC patients who receive surgery and chemotherapy

**Fig. 2:**
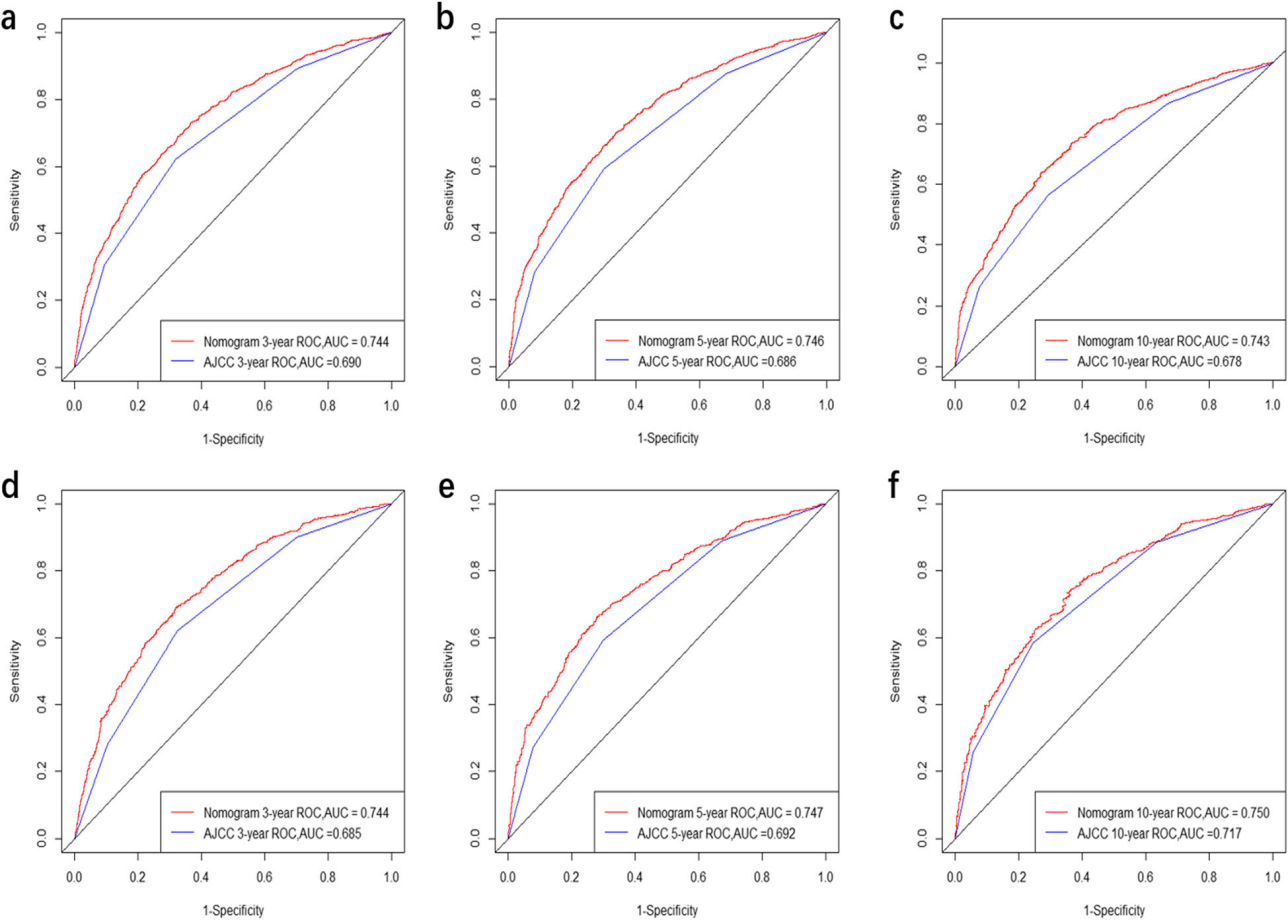
ROC curves. The ability of the model to be measured by the C index. **a, b, c** 3-,5-, 10-year CSS came from the training set, and **d, e, f** 3-,5-, 10-year CSS came from the validation set

The constructed nomogram was externally validated using the validation set. The predictive capacity of the constructed nomogram was directly contrast the seventh edition of AJCC staging system for GC. The C-indexes for the training and validation sets were larger for the nomogram (0.694 and 0.707, respectively) than for the seventh AJCC staging system (0.651 and 0.661). The 3-, 5-, and 10-year AUCs for the nomogram were 0.744, 0.746, and 0.743, respectively, for the training set, and 0.744, 0.747, and 0.75 for the validation set, indicating a good model discrimination ability that was better than that of the seventh AJCC staging system (Fig. 2).

**Fig. 3:**
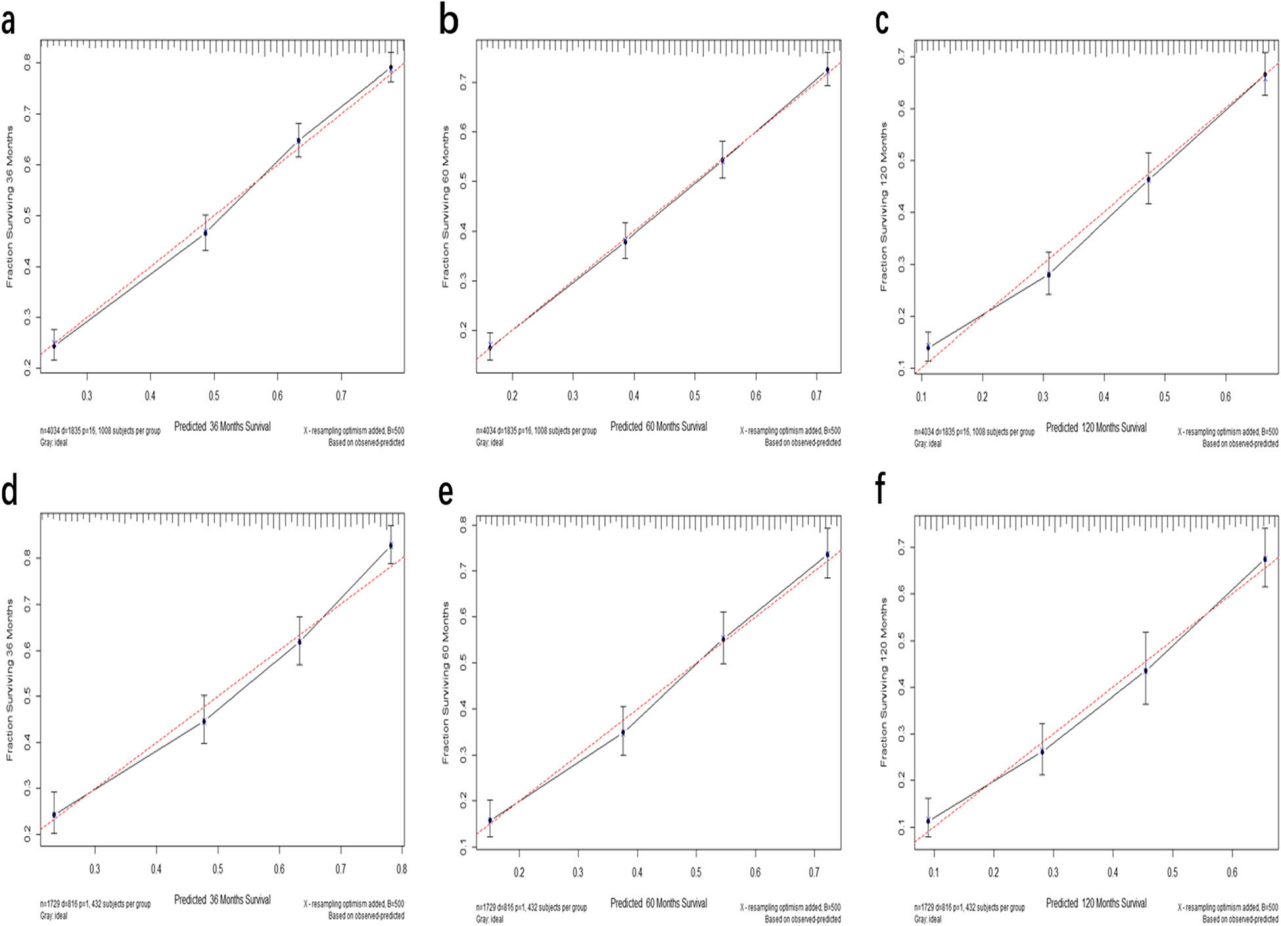
Calibration plots. Show the relationship between the predicted probabilities base on the nomogram and actual values of the train set (**a**, **b**, **c**) and validation set (**d**, **e**, **f**)

Calibration plots for the proposal nomogram displayed that the predicted 3-, 5-, and 10-year DSS probabilities for the training and validation sets of patients from SEER database were almost identical to the actual observations (Fig. 3).

The NRI values for the 3-, 5-, and 10-year DSS were 0.380 (95% CI = 0.316–0.470), 0.407 (95% CI = 0.350–0.505), and 0.413 (95% CI = 0.336–0.519), respectively, in the validation set. These results displayed that the proposed nomogram presented a large improvement in predictive performance. Similarly, the IDI values for the 3-, 5-, and 10-year DSS were 0.058, 0.059, and 0.058, respectively, in the validation set (*P* < 0.05), further validating the improved predictive performance of the nomogram.

### Decision curve analysis

**Fig. 4:**
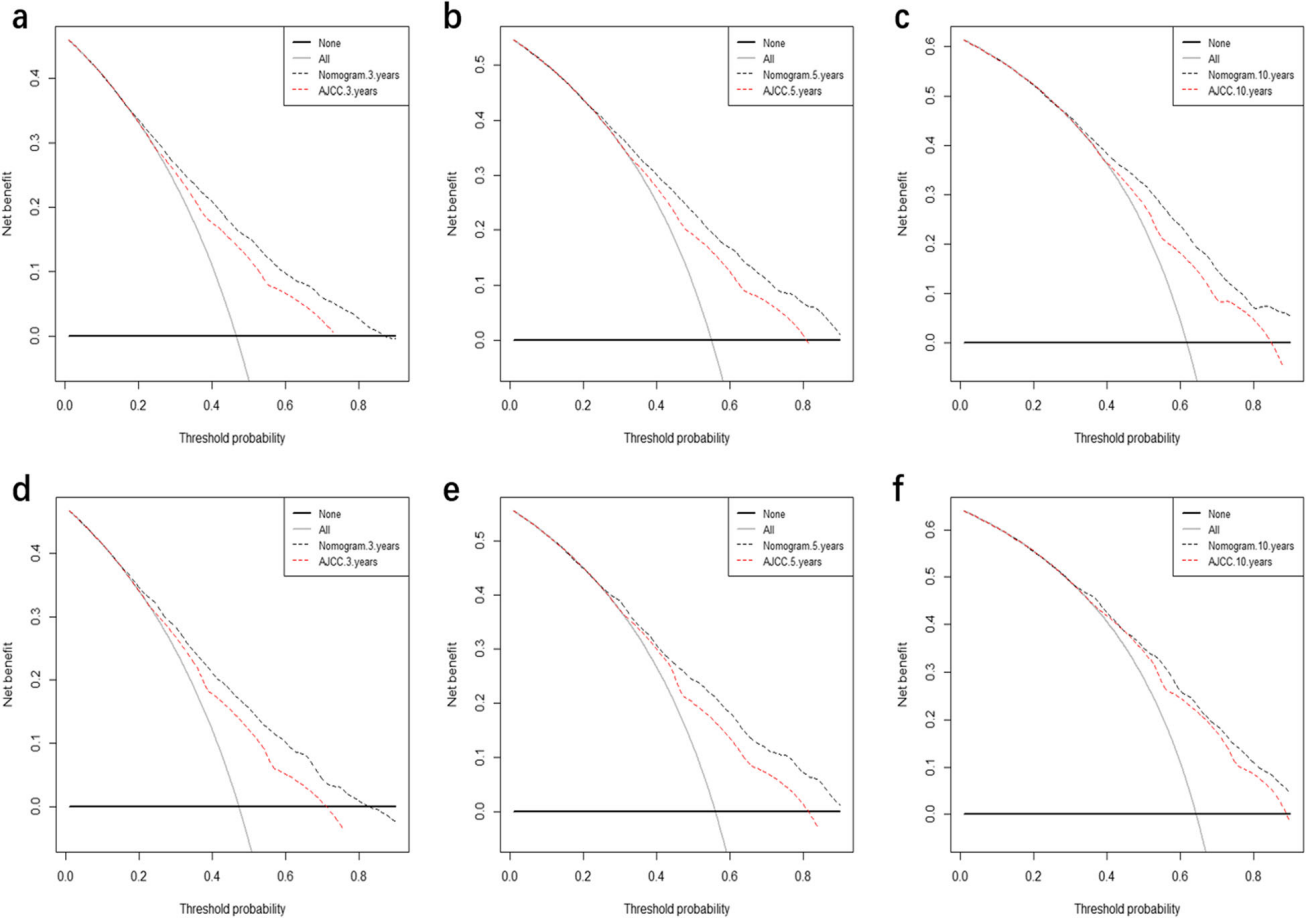
Decision curve analysis. In the figure, the abscissa is the threshold probability, the ordinate is the net benefit rate. The horizontal one indicates that all samples are negative and all are not treated, with a net benefit of zero. The oblique one indicates that all samples are positive. The net benefit is a backslash with a negative slope. **a**, **b**, and **c** came from the training set; and **d**, **e** and **f** came from the validation set

DCA plots for the 3-, 5-, and 10-year DSS discrimination ability are depicted in Fig. 4. The proposed nomogram was found to consistently perform better than the seventh AJCC staging system.

## Discussion

This study developed a nomogram to predict the 3-, 5-, and 10-year DSS of GAC patients who receive surgery and chemotherapy based on multi-center and multi-population and multi-ethnic data in the SEER database. The AJCC staging system has been the most commonly used and most-effective program for predicting the prognosis of GAC patients [20]. However, receiving both surgery and chemotherapy will result in many more important risk factors influencing the DSS, such as age, race, sex, marital status, primary cancer site, grade, and summary stage. We therefore implemented a more-comprehensive prognosis model in the form of a nomogram. This nomogram not only includes the AJCC staging system, but also system demographics and other important clinical parameters. The overall survival of GAC patients is prolonged after they receive surgery and chemotherapy [21–24]. The increasing number of GAC survivors—especially long-term survivors—makes the use of a nomogram for predicting their long-term survival prognosis highly desirable.

To our knowledge, the present nomogram is the first for predicting the 10-year DSS for GAC patients who receive surgery and chemotherapy. Zhong and colleagues constructed a similar nomogram for the 10-year survival prognosis of GC patients after they receive curative surgery, but that nomogram did not include the chemotherapy status [25]. In addition, an external cohort of GAC patients from the same database was used to validate the present nomogram. The obtained results suggest that we have successfully constructed a reliable nomogram for predicting the 3-, 5-, and 10-year DSS of GAC patients who receive surgery and chemotherapy, since the nomogram validation demonstrated favorable discrimination and calibration.

The constructed nomogram includes several independent prognostic factors. Many studies have indicated that the age of cancer patients is an important prognostic factor for DSS [13, 26–28]. Multivariate analyses indicated that the RNP and older age were statistical independent risk factors for the DSS of GAC patients who receive surgery and chemotherapy, with survival being worse in older patients. The current study found that the DSS of GAC patients who receive surgery and chemotherapy was negatively correlated with age. Moreover, the race that not white or black appeared to be a protective factor compared with being white (HR = 0.765, 95% CI = 0.668–0.877, *P* < 0.001), while cancer with a primary site of the pylorus seemed to be protective compared with the cardia(HR = 0.729, 95% CI = 0.649–0.818, *P* < 0.001). Undifferentiated classification was a risk factor compared with well-differentiated (HR = 1.581, 95% CI = 1.026–2.437, *P* < 0.001), a higher AJCC stage was associated with a worse DSS, and compared with a localized summary stage, a distant summary stage was a risk factor for DSS (HR = 1.726, 95% CI = 1.263–2.659, *P* < 0.001).

The proposed predicting nomogram model includes risk factors that are easily gotten and collected through clinical historical records. To further estimate whether the prognostic nomogram model expressed better than the traditional AJCC staging system, we assessed its performance based on calibration, discrimination, IDI, NRI, and DCA. The proposed nomogram displayed a good discrimination ability by producing a C-index of 0.694 for the training set and 0.707 for the validation set. Moreover, the C-indexes of the AJCC staging system were weaker than those of the proposed nomogram, as were the AUC values. The discriminative efficiency of the nomogram was obviously preferably comparing with that of the AJCC staging system. The plots for both the training set and validation set resembled a 45-degree line, showing that the predictions of the proposed nomogram were well calibrated (Fig. 2). The NRI and IDI are more sensitive indicators than the C-index, and the NRI indicated that the proposed model reclassified the risk probabilities better than did the AJCC staging system, while the IDI demonstrated the superior ability of the constructed nomogram to distinguish cases compared with the AJCC staging system. Numerous previous studies have found benefits of applying DCA [29–33], and the results of the current study showed that the 3-, 5-, and 10-year DCA curves displayed net benefits larger than those of the AJCC staging system, both in the training set and validation set (Fig. 4).

While the present nomogram model demonstrated high accuracy in predicting DSS, several limitations of this study must be considered. Firstly, data of the patients were collected from the SEER database, in which the chemotherapy status is only reported as either “yes” and “no/unknown.” Although all of the cases included in the current study were GAC patients who had received chemotherapy (by excluding the “no/unknown” ones in the SEER database), the lack of detailed chemotherapy information might have influenced the obtained results. Secondly, there are potentially other factors that could influence the prognosis of GAC cancer patients, and so further clinical research should be carried out to improve the nomogram. Thirdly, since the current study had a retrospective research type design, some important data of including patients might had been missing inevitably, decreasing the number of eligible cases. Fourthly, the results of this study would be more meaningful if the nomogram model was well externally validated by another real world, independent, large-quantity, high-quality cohort, which would prove whether our findings are more-widely acceptable. Although there are so many limitations, The results show that our prognostic nomogram is an instructive and efficient model to predict the accurate individual survival outcomes of GAC patients who receive surgery and chemotherapy.

## Conclusion

We have constructed and validated a prognosis nomogram for GAC that has a high accuracy. The prognostic effect of the proposed nomogram was better than that of the traditional seventh AJCC staging system alone. The validation process indicated that the current nomogram provides more efficient DSS predictions for GAC patients who receive surgery and chemotherapy in the general population. The nomogram will be beneficial for personalized survival prediction and helpful in clinical decision-making for GAC patients who receive surgery and chemotherapy.

## Data Availability

The dataset from SEER database generated and/or analyzed during the current study are available in the SEER dataset repository (https://seer.cancer.gov/).

## Abbreviations

AC: Adenocarcinoma
AJCC: American Joint Committee on Cancer
AUC: Area under the receiver operating characteristics curve
CI: Confidence interval
C-index: Consistency index
CSS: Cancer-specific survival
DCA: Decision curve analysis
DSS: Disease-specific survival
DSW: Divorced & separated &widowed
GAC: Gastric adenocarcinoma
HR: Hazard ratio
ICD-O-3: The third revision of International Classification of Disease for Oncology
OS: Overall survival
PUC: Primary urethral carcinoma
RNE: Number of regional nodes examined
RNP: Number of regional nodes positive
SCC: Squamous cell carcinoma
SEER: Surveillance, Epidemiology, and End Results
Sums: SEER Summary stage 2000
TCC: Transitional cell carcinoma
